# Case report: Childhood epilepsy and borderline intellectual functioning hiding an AADC deficiency disorder associated with compound heterozygous *DDC* gene pathogenic variants

**DOI:** 10.3389/fneur.2023.1284339

**Published:** 2023-12-05

**Authors:** Ida Cursio, Sabrina Siliquini, Claudia Carducci, Giovanni Bisello, Mario Mastrangelo, Vincenzo Leuzzi, Mariarita Bertoldi, Carla Marini

**Affiliations:** ^1^Child Neurology and Psychiatric Unit, Pediatric Hospital G. Salesi, Azienda Ospedaliero Universitaria delle Marche, Ancona, Italy; ^2^Department of Experimental Medicine, Sapienza - Università di Roma, Rome, Italy; ^3^Department of Neuroscience, Biomedicine and Movement Sciences, University of Verona, Verona, Italy; ^4^Department of Women/Child Health and Urological Science, Sapienza - Università di Roma, Rome, Italy; ^5^Department of Human Neuroscience, Sapienza - Università di Roma, Rome, Italy

**Keywords:** epilepsy, focal seizures, AADC deficiency, *DDC* gene, compound heterozygous variants, autonomic dysfunction

## Abstract

Aromatic L-amino acid decarboxylase (AADC) deficiency is a rare autosomal recessive neurometabolic disorder leading to severe combined serotonin, dopamine, norepinephrine, and epinephrine deficiency. We report on a female patient with borderline functioning and sporadic clear-cut focal to bilateral seizures from age 10 years. A neuropsychological assessment highlighted a mild impairment in executive functions, affecting attention span and visual–spatial abilities. Following the diagnosis of epilepsy with a presumed genetic etiology, we applied a diagnostic approach inclusive of a next-generation sequencing (NGS) gene panel, which uncovered two variants *in trans* in the DOPA decarboxylase (*DDC*) gene underlying an AADC deficiency. This compound heterozygous genotype was associated with a mild reduction of homovanillic acid, a low level of the norepinephrine catabolite, and a significant reduction of 5-hydroxyindoleacetic acid in cerebrospinal fluid. Remarkably, 3-O-methyldopa (3-OMD) and 5-hydroxytryptophan were instead increased. During the genetically guided re-evaluation process, some mild signs of dysautonomic dysfunction (nasal congestion, abnormal sweating, hypotension and fainting, excessive sleepiness, small hands and feet, and increased levels of prolactin, tiredness, and fatigue), more typical of AADC deficiency, were evaluated with new insight. Of the two AADC variants, the R347Q has already been characterized as a loss-of-function with severe catalytic impairments, while the novel L391P variant has been predicted to have a less severe impact. Bioinformatic analyses suggest that the amino acid substitution may affect affinity for the PLP coenzyme. Thus, the genotype corresponds to a phenotype with mild and late-onset symptoms, of which seizures were the clinical sign, leading to medical attention. This case report expands the spectrum of AADC deficiency phenotypes to encompass a less-disabling clinical condition including borderline cognitive functioning, drug-responsive epilepsy, and mild autonomic dysfunction.

## Introduction

Aromatic L-amino acid decarboxylase (AADC) deficiency (OMIM 608643) is a rare, autosomal recessive, inborn error of neurotransmitter biosynthesis resulting in a combined deficiency of serotonin, dopamine, and the metabolic derivatives norepinephrine and epinephrine (1).

AADC deficiency is caused by biallelic pathogenic variants in the *DDC* gene, and most identified genotypes are compound heterozygous (73%) (2). In the last few years, there has been a marked increase in the number of identified variants, yet no clear genotype–phenotype correlation has been identified.

*The DDC* gene, located on chromosome 7p12.2-p12.1, is a protein-coding gene encoding the AADC enzyme that is implicated in two important metabolic pathways of neurotransmitter synthesis. It enables 5-hydroxy-L-tryptophan decarboxylase activity to form serotonin from 5-OH tryptophan and L-DOPA decarboxylase activity, giving rise to dopamine from L-dihydroxyphenylalanine (L-DOPA) (3). AADC deficiency presents with an infantile encephalopathy resulting in severe neurological and neurodevelopmental impairment, leading to a permanent, severe disabling condition in approximately 80% of patients (4).

Presenting symptoms of AADC deficiency include movement disorders (oculogyric crisis, dystonia, and hypokinesia), hypotonia, developmental delay, pseudo-myasthenic features (ptosis and fatigability), and autonomic dysfunction (nasal congestion, abnormal sweating, excessive drooling, orthostatic hypotension, bradycardia, and temperature instability). Less common symptoms are episodes of hypoglycemia, prematurity, failure to thrive, behavioral and sleep disorders, and gastrointestinal symptoms (gastroesophageal reflux, diarrhea, and constipation) (5–7). Epilepsy associated with AADC deficiency is a rare finding described only in 4–7% of published patients (5, 6).

Clinical diagnosis is based on the genetic analysis of *DDC* confirmed by assessing dopamine and serotonin metabolites in CSF. Blood and CSF levels of 3-*O*-methyldopa (3-OMD) and blood prolactin may be useful surrogate diagnostic biomarkers (5, 6).

Treatment options include oral pyridoxine, dopamine-mimetic drugs, and inhibitors of dopamine catabolism (5). Intracerebral gene therapy has been recently developed as an alternative treatment for severely affected patients (8–14).

In this study, we describe a 13-year-old girl with late-onset, mild, and atypical AADC deficiency diagnosed ‘by chance’ with a next-generation sequencing (NGS) multigene panel, which included the *DDC* gene.

## Case report

### Clinical history

A 13-year-old female of Caucasian ethnic origin originated from a small town in the north-west area of Sicily. The father had epilepsy in childhood (responsive to carbamazepine, discontinued after a few years), and the mother had migraine; two siblings were healthy.

Psychomotor developmental milestones were reached at the correct age; past medical history was uneventful but for sporadic migraine episodes since childhood and sleep apnea with snoring.

At the age of 10 years, during wakefulness, she presented an episode of loss of awareness, head and eye deviation to the right, and gestural automatisms lasting a few minutes, followed by gradual, spontaneous recovery. The second episode occurred 4 months later, during sleep, when she was found unconscious with bilateral clonic jerking. EEG recording, performed soon after the seizure, reported multifocal spikes and sharp waves. She was treated with levetiracetam at a dose of 750 mg/daily. After 2 years without seizures, levetiracetam was gradually stopped. Yet, 3 months after discontinuing the medication, seizures relapsed, and treatment was restarted. On clinical examination, she showed hands and feet smaller than expected, mild bilateral ptosis, mild bradykinesia, and clumsiness in gross motor tasks. Deep tendon reflexes were normal. No psychiatric comorbidities emerged from clinical observation and anamnestic interview with the girl However, her parents described her as shy and avoidant of social interaction with peers. Cognitive assessment (*Wechsler Intelligence Scale for Children - IV*) revealed low normal performances (total IQ score 78) with a disharmonic profile for prevalent perceptual reasoning index impairment (score: 76) with respect to working memory (score: 88), verbal comprehension (score: 86), and processing speed (score: 82). Overall, the neuropsychological assessment highlighted a mild impairment in executive functions, mainly affecting attention span and visual–spatial abilities.

**Figure 1:**
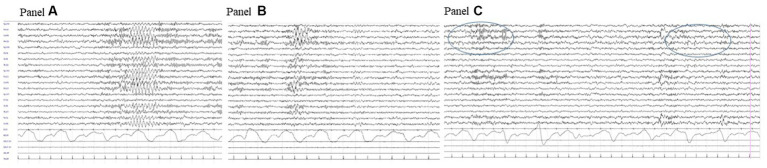
Interictal video-EEG recording at age 11 years: polygraphic recording during drowsiness showing a bilateral discharge of rhythmic sharply contoured delta waves **(A)**; a similar activity but with right predominance **(B)** and right occipital spikes during sleep (circled area, **C**).

**Figure 2:**
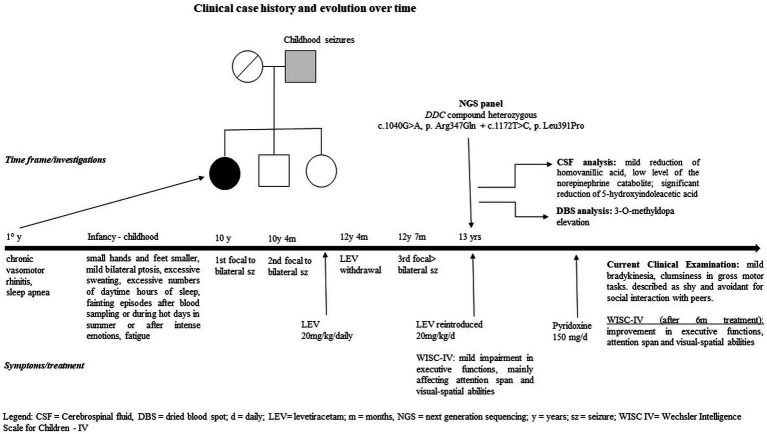
Clinical case history and evolution over time, including diagnostic procedures.

Brain CT and MRI scans were normal. Current, interictal video-EEG monitoring showed sporadic sharp waves in the occipital bilateral regions during sleep (Figure 1). The clinical history of the patient, evolution over time, and applied diagnostic procedures are summarized in Figure 2.

We then applied a NGS approach used in many gene identification procedures (15–18) by exploiting a commercial gene panel. In detail, a saliva sample was collected and sent to Blueprint Genetics Oy (Keilaranta 16 A-B, 02150 Espoo, Finland) for NGS analysis. Blueprint Genetics FLEX Comprehensive Epilepsy Panel Plus (covering 668 genes and 9,999 exons, version 1, May 17, 2022) was used. The test was developed, approved, and certified as reported in the Manufacturer’s Report (ISO 15189 accreditation). Details of the process and quality control systems for identifying the candidate variants are provided in Supplementary methods from the Manufacturer Datasheet.

The sequence analysis detected two missense variants on the *DDC* gene of paternal (NM000790.4, c.1040G > A, p. Arg347Gln) and maternal (NM000790.4, c.1172 T > C, p. Leu391Pro) origin. The Arg347Gln amino acid transition was reported as pathogenic by ClinVar and has already been reported in many patients with AADC deficiency (2, 4). The Leu391Pro substitution is novel and classified as a variant of uncertain significance.

#### Bioinformatic analysis of the AADC

The human AADC structure predicted by AlphaFold2 (AF2) (19) was used to determine the position of Leu391, and the absence of cofactors in the AF2 predicted proteins has been overcome by the algorithm AlphaFill (20). Since AADC is an obligate functional dimer, we determined the impact of amino acid substitution on the AADC protein population that theoretically can be synthesized by the compound heterozygous patient. Three different types of AADC dimers protein could indeed be present: the homodimers of Arg347Gln and Leu391Pro variants and the Arg347Gln/Leu391Pro heterodimer (21–23). The Arg347Gln homodimeric variant has already been characterized as loss-of-function with severe catalytic impairments and classified as pathogenic (2).

**Figure 3:**
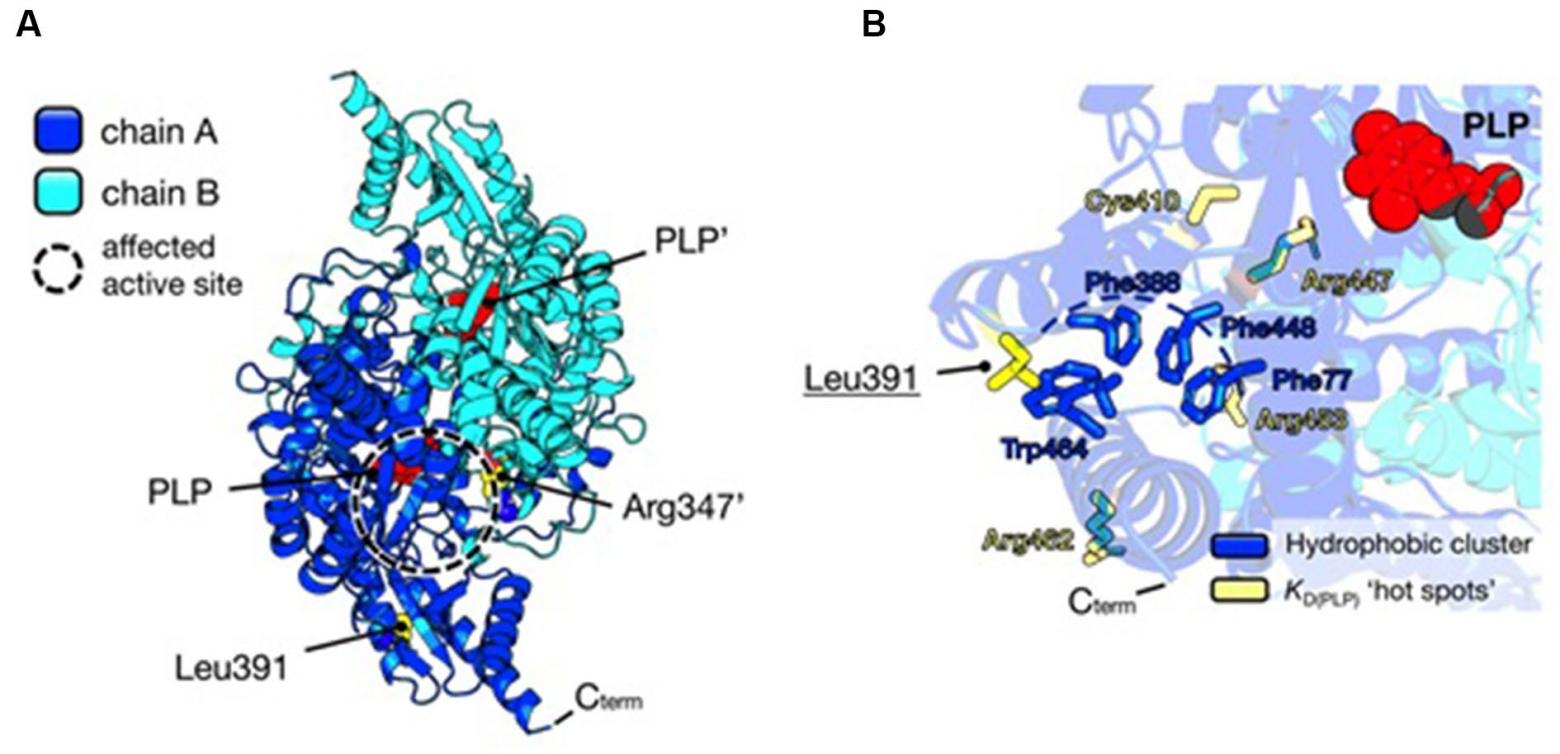
Structural analysis of AADC protein and localization of substituted amino acids. **(A)** AADC dimeric structure is represented as a cartoon. Chain A is blue and Chain B is cyan. PLP molecule for each active site is represented as red spheres. Leu391 and Arg347’ in the heterodimeric protein are shown as yellow spheres. The prime indicates the residue and cofactor molecules belonging to subunit B. The active site where both variants may exert their effect is highlighted as a dashed circle. **(B)** Localization of Leu391. The cluster of hydrophobic residues (Phe77, Phe388, Phe448, and Trp464) shielded from the solvent is shown as blue sticks, while yellow sticks represent residues whose pathogenic substitutions lead to decreased affinity for the PLP molecule (red spheres). Protein visualization, measurement, dimer building, and rotamer analysis were performed using PyMOL 2.2.3, The PyMOL Molecular Graphics 50 System, Version 2.0 Schrödinger, LLC, New York, NY, 2021.

Instead, the Leu391Pro variant is a novel and never-characterized substitution. *In silico* inspection of the AADC crystal structure shows that Leu391 is located on a surface alpha-helix of the C-terminal domain distant from the active site (Figure 3A), possibly involved in hydrophobic cluster stabilization important for folding and pyridoxal 5′-phosphate (PLP) binding (Figure 3B), since other pathogenic variants (Glu283Ala, Cys410Gly, Arg447His, Arg453Cys, and Arg462Gln) mapping in this protein region affect affinity for the coenzyme (24–26). Its substitution for Pro may destabilize the helix, leading to the exposure of hydrophobic residues and somewhat influencing PLP binding. Interestingly, it can be predicted (2) that the AADC heterodimer maintains one active site fully functional while the other is affected by both Arg347Gln and Leu391Pro substitutions.

#### Clinical reassessment following genetic or metabolic diagnosis

Following the genetic result, we interviewed the father, focusing on all signs/symptoms associated with AADC deficiency. He reported that the daughter tended to sleep several hours during the day; she also easily fainted after blood sampling, during hot days in the summer, or after intense emotions. She has suffered from excessive sweating and chronic vasomotor rhinitis since her first years of life. Finally, she complained of lower limb pain after prolonged inactivity. Table 1 highlights clinical findings in our patient compared with a typical AADC presentation.

**Table 1:** Laboratory analysis of liquor neurotransmitters metabolites.

Liquor neurotransmitters metabolites		
Neurotransmitter	Result (nmol/L)	Reference values (nmol/L)
Homovanillic acid	101	148–434
5-hydrosyindoleacidic acid	7	68–115
3-O-metildopa	542	<50
5-hydroxytryptophan	45	<10

CSF examination showed a mild reduction of homovanillic acid (101 nmol/L; r.v. 148–434), an incongruently low level of the norepinephrine catabolite 3-methoxy-4-hydroxyphenylglycol (11 nmol/L; r.v. 28–60), and a more remarkable reduction of 5-hydroxyindoleacetic acid (7 nmol/L; r.v. 68–115). As expected, 3-OMD (542 nmol/L (r.v. < 50)) and 5-hydroxytryptophan (45 nmol/L (r.v. <10)) were increased (Table 2). 3-OMD elevation was also found in dried blood spot (DBS) (2,820 nmol/L;r.v. <1,000 nmol/L) (Table 1) (15), while prolactin was marginally increased (27.2 ng/mL, r.v. 27 ng/mL).

**Table 2:** Clinical findings in our patient compared with typical AADC presentation.

Clinical findings	
Sign / symptoms of AADC deficiency	Patient
**Common**	
Early onset hypotonia	
Developmental delay	x
Oculogyric crisis	
Dystonia	
Hypokinesia	
Nasal congestion	x
Abnormal sweating	x
Excessive drooling	
Hypotension	x
Bradycardia	
Temperature instability	
Pseudo-myasthenic features (ptosis and fatigue)	x
**Less common**	
Infantile episodes of hypoglycemia	
Behavioral disorders	
Insomnia	
Excessive sleepiness	x
Gastrointestinal symptoms	
Epilepsy	x
**Other findings**	
Hyperreflexia	
Small hands and feet	x
Sleep apnea	x

The patient is currently being treated with pyridoxine 150 mg/daily and levetiracetam 1,500 mg/daily. Since she started treatment, she no longer fainted, while sleep disturbances and abnormal sweating remained unchanged. Neuropsychological re-evaluation after 6 months of pyridoxine supplementation showed an improvement in executive functions and overall cognitive performance at WISC-IV.

## Discussion

We report on a 13-year-old patient reaching a diagnosis of AADC deficiency. Childhood epilepsy with recurring focal to bilateral seizures and borderline intellectual functioning with mild impairment of the executive functions affecting attention span and visual–spatial abilities were the clinical signs leading the girl to medical attention, thus uncovering a phenotype that is not typically related to AADC deficiency.

The current diagnostic approach to patients with unexplained epilepsy and variable degrees of intellectual disability includes the application of NGS gene panels, which in our patient led to the unexpected result of compound heterozygous variants of the *DDC* gene. A subsequent targeted clinical interview uncovered some features attributable to AADC deficiency. We regarded with new insight some clinical aspects, including nasal congestion, sleep apnea, frequent faints, abnormal sweating, a slow and awkward bradykinetic attitude, small hands and feet, and emotional lability. We also noticed that many of the Italian patients affected by AADC deficiency come from Sicily (4, 27), a populated island in the Mediterranean; differently from what was reported in Taiwan (28), a founder effect could not be identified.

Most signs and symptoms described in patients with AADC deficiency can be attributed to dopamine, norepinephrine, epinephrine, and serotonin deficiencies. Disturbance in serotonin biosynthesis affects appetite, sleep, memory, learning, body temperature, mood, cardiovascular function, and endocrine functions. In our patient, the prevalence of dysautonomic symptoms is consistent with a prevalent deficiency of norepinephrine and serotonin and a relatively preserved dopamine synthesis. Indeed, the mild reduction of HVA (the surrogate marker of dopamine) and the low concentration of CSF MHPG (the surrogate marker of norepinephrine) cannot be attributed to enzyme-substrate depletion. The high frequency in the general population of polymorphic variants on the dopamine beta-hydroxylase gene associated with variable reductions in enzymatic activity might contribute to this discrepancy (29). We could also speculate that the mild motor symptoms observed in our patients might be related to a progressive autoregulation of the dopaminergic (but not serotoninergic) network occurring with age and resulting in a progressive increase of brain dopamine levels via pre- and postsynaptic adaptive mechanisms, as observed in murine models (4, 30).

Epilepsy is a rare finding in AADC patients, reported in only 7.6% (9 of 117 patients) examined for the development of AADC guidelines (5) and in none of the 64 patients reported by Pearson et al. (7). EEG abnormalities, including slowing, fast activity, and poly-spikes, have been reported in some patients (6, 31). We can argue that the decreased cerebral serotoninergic system might contribute to the epilepsy that was our patient’s presenting and more relevant clinical signs. Indeed, it is known that depletion of brain 5-HT lowers the threshold for audiogenically, chemically, and electrically evoked convulsions (32). There is also ample preclinical and clinical evidence suggesting the importance of serotonergic neurotransmission in human epilepsy. Current research highlights the potential of modulating serotonergic transmission and targeting distinct serotonin (5-HT) receptors in treating epilepsy (33).

Our proband carries compound heterozygous pathogenic variants combining a known splice site catalytic variant with severe functional impact with a novel missense change with minor functional consequences. A positive or negative complementation has been proposed in compound heterozygotes based on the number of affected active sites in heterodimeric AADC proteins (2, 21). In this case, the milder mutation in the heterodimer could positively complement the most severe one, since only one active site is negatively affected while the other can properly function. These data agree with the patient’s mild phenotype. Indeed, bioinformatic analysis suggests that the Leu391Pro variant may affect affinity for the coenzyme (PLP), which is confirmed by the improvement the patients experienced after 6 months of B6 treatment. Yet, since the Arg347Gln variant is catalytically affected, treatment with vitamin B6 cannot fully rescue the phenotype. As a result of systematic clinical observation, non-ergot dopamine agonists, such as rotigotine and pramipexole, inhibitors of MAO and/or COMT, might be considered as potential treatments (4, 34, 35). The patient showed a good response to monotherapy with B6 supplementation; thus, further therapeutic decisions will be based on a clinical follow-up and, possibly, a new CSF dosage of biogenic amine metabolites.

AADC has been the first genetic disease for which effective intracerebral gene therapy has been developed. This treatment has dramatically improved the neurological defect related to dopamine depletion (8–13). *DDC* gene therapy is currently a valid option for patients unresponsive to pharmacological therapy. It is approved in the European Union and United Kingdom for patients older than 18 months with severe motor and developmental impairment (14).

In conclusion, the present case enlarges the phenotypic spectrum of AADC deficiency, encompassing less-disabling conditions, such as borderline cognitive functioning, drug-responsive epilepsy, and prevalent autonomic dysfunction. This case report also highlights how heterogeneous this condition is, thus underlying the probability that it might still be underdiagnosed.

## Data availability statement

The datasets presented in this study can be found in online repositories. The names of the repository/repositories and accession number(s) can be found in the article/Supplementary material.

## Ethics statement

The studies involving humans were approved by Comitato etico territoriale delle marche; azienda ospedaliera universitaria (AOU) marche, Ancona, Italy. The studies were conducted in accordance with the local legislation and institutional requirements. Written informed consent for participation in this study was provided by the participants’ legal guardians/next of kin. Written informed consent was obtained from the individual(s) for the publication of any potentially identifiable images or data included in this article.

## Author contributions

IC: Conceptualization, Investigation, Data curation, Methodology, Writing – original draft, Writing – review & editing. SS: Conceptualization, Investigation, Writing – original draft, Writing – review & editing, Data curation, Methodology. CC: Data curation, Investigation, Methodology, Writing – review & editing. GB: Data curation, Methodology, Writing – review & editing, Investigation. MM: Data curation, Methodology, Writing – review & editing, Conceptualization. VL: Conceptualization, Data curation, Methodology, Writing – review & editing, Supervision. MB: Data curation, Methodology, Supervision, Investigation, Writing – original draft, Writing – review & editing. CM: Investigation, Data curation, Methodology, Supervision, Writing – original draft, Conceptualization, Writing – review & editing.
