# Supplementary material for: Case report: Childhood epilepsy and borderline intellectual functioning hiding an AADC deficiency disorder associated with compound heterozygous DDC gene pathogenic variants
Source: Front Neurol. 2023 Dec 5;14:1284339. doi: 10.3389/fneur.2023.1284339 (PMC10729769; doi:10.3389/fneur.2023.1284339)
Supplement: Supplementary file 1 [file Data_Sheet_1.docx]

**Supplementary Methods**

**Process of identification of *DDC* variants from Blueprint Genetics Manufacturer Datasheet.**

Analysis was carried out by Blueprint Genetics Oy (Keilaranta 16 A-B, 02150 Espoo, Finland). Laboratory process: When required, the total genomic DNA was extracted from the biological sample using bead-based method. DNA quality and quantity were assessed using electrophoretic methods at Blueprint Genetics. After assessment of DNA quality, qualified genomic DNA sample was randomly fragmented using non-contact, isothermal sonochemistry processing. Sequencing library was prepared by ligating sequencing adapters to both ends of DNA fragments. Sequencing libraries were size-selected with bead-based method to ensure optimal template size and amplified by polymerase chain reaction (PCR). Regions of interest (exons and intronic targets) were targeted using hybridization-based target capture method. The quality of the completed sequencing library was controlled by ensuring the correct template size and quantity and to eliminate the presence of leftover primers and adapter-adapter dimers. Ready sequencing libraries that passed the quality control were sequenced using the Illumina's sequencing-by-synthesis method using paired-end sequencing (150 by 150 bases). Primary data analysis converting images into base calls and associated quality scores was carried out by the sequencing instrument using Illumina's proprietary software, generating CBCL files as the final output. These steps were performed at Blueprint Genetics.

Bioinformatics and quality control: Base called raw sequencing data was transformed into FASTQ format using Illumina's software (bcl2fastq). Sequence reads of each sample were mapped to the human reference genome (GRCh37/hg19). Burrows-Wheeler Aligner (BWA-MEM) software was used for read alignment. Duplicate read marking, local realignment around indels, base quality score recalibration and variant calling were performed using GATK algorithms (Sentieon) for nDNA. Variant data for was annotated using a collection of tools (VcfAnno and VEP) with a variety of public variant databases including but not limited to gnomAD, ClinVar and HGMD. The median sequencing depth and coverage across the target regions for the tested sample were calculated based on MQ0 aligned reads. The sequencing run included in-process reference sample(s) for quality control, which passed our thresholds for sensitivity and specificity. The patient's sample was subjected to thorough quality control measures including assessments for contamination and sample mix-up. Copy number variations (CNVs), defined as single exon or larger deletions or duplications (Del/Dups), were detected from the sequence analysis data using a proprietary bioinformatics pipeline. The difference between observed and expected sequencing depth at the targeted genomic regions was calculated and regions were divided into segments with variable DNA copy number. The expected sequencing depth was obtained by using other samples processed in the same sequence analysis as a guiding reference. The sequence data was adjusted to account for the effects of varying guanine and cytosine content. Bioinformatics and quality control processes were performed by Blueprint Genetics.

Interpretation: The clinical interpretation team assessed the pathogenicity of the identified variants by evaluating the information in the patient requisition, reviewing the relevant scientific literature and manually inspecting the sequencing data if needed. All available evidence of the identified variants was compared to classification criteria. Reporting was carried out using HGNC-approved gene nomenclature and mutation nomenclature following the HGVS guidelines. Likely benign and

benign variants were not reported. The interpretation was performed at Blueprint Genetics.

Variant classification: Our variant classification follows the Blueprint Genetics from the ACMG guideline 2015. Minor modifications were made to increase reproducibility of the variant classification and improve the clinical validity of the report. The classification and interpretation of the variant(s) identified reflect the current state of Blueprint Genetics’ understanding at the time of this report. Variant classification and interpretation are subject to professional judgment, and may change for a variety of reasons, including but not limited to, updates in classification guidelines and availability of additional scientific and clinical information. This test result should be used in conjunction with the health care provider's clinical evaluation. Inquiry regarding potential changes to the classification of the variant is strongly recommended prior to making any future clinical decision.

Databases: The pathogenicity potential of the identified variants were assessed by considering the predicted consequence of the change, the degree of evolutionary conservation as well as the number of reference population databases and mutation databases such as, but not limited to, the gnomAD, ClinVar, HGMD Professional and Alamut Visual. In addition, the clinical relevance of any identified CNVs was evaluated by reviewing the relevant literature and databases such as Database

of Genomic Variants and DECIPHER. For interpretation of mtDNA variants specific databases including e.g. Mitomap, HmtVar and 1000G were used.

Confirmation of sequence alterations: Sequence variants classified as pathogenic, likely pathogenic and variants of uncertain significance (VUS) were confirmed using bi-directional Sanger sequencing when they did not meet our stringent NGS quality metrics for a true positive call. In addition, prenatal case with diagnostic findings were confirmed. The confirmation of sequence alterations was performed at Blueprint Genetics.

Confirmation of copy number variants: CNVs (Deletions/Duplications) were confirmed using a digital PCR assay if they covered less than 10 exons (heterozygous), less than 3 exons (homo/hemizygous) or were not confirmed at least three times previously at our laboratory. Furthermore, CNVs of any size were not confirmed when the breakpoints of the call could be determined. The confirmation of copy number variants was performed at Blueprint Genetics.

Analytic validation: The detection performance of this panel is expected to be in the same range as our high-quality, clinical grade NGS sequencing assay used to generate the panel data (nuclear DNA: sensitivity for SNVs 99.89%, indels 1-50 bps 99.2%, one-exon deletion 100% and five exons CNV 98.7%, and specificity >99.9% for most variant types). It does not detect very low-level mosaicism as a variant with minor allele fraction of 14.6% can be detected in 90% of the cases.

Detection performance for mtDNA variants (analytic and clinical validation): sensitivity for SNVs and INDELs 100.0% (10-100% heteroplasmy level), 94.7% (5-10% heteroplasmy level), 87.3% (<5% heteroplasmy level) and for gross deletions 100.0%. Specificity is >99.9% for all.

Test restrictions: A normal result does not rule out the diagnosis of a genetic disorder since some DNA abnormalities may be undetectable by the applied technology. Test results should always be interpreted in the context of clinical findings, family history, and other relevant data. Inaccurate, or incomplete information may lead to misinterpretation of the results.

Technical limitations: This test does not detect the following: complex inversions, gene conversions, balanced translocations, repeat expansion disorders unless specifically mentioned, non-coding variants deeper than ±20 base pairs from exon-intron boundary unless otherwise indicated (please see the list of non-coding variants covered by the test). Additionally, this test may not reliably detect the following: low level mosaicism, stretches of mononucleotide repeats, indels

larger than 50bp, single exon deletions or duplications, and variants within pseudogene regions/duplicated segments. The sensitivity of this test may be reduced if DNA is extracted by a laboratory other than Blueprint Genetics. Laboratory error is also possible.

Regulation and accreditations: This test was developed and its performance characteristics determined by Blueprint Genetics. It has not been cleared or approved by the US Food and Drug Administration. This analysis has been performed in a CLIA-certified laboratory (#99D2092375), accredited by the College of American Pathologists (CAP #9257331) and by FINAS Finnish Accreditation Service, (laboratory no. T292), accreditation requirement SFS-EN ISO 15189:2013. All the tests are under the scope of the ISO 15189 accreditation.
